# TMIGD1 acts as a tumor suppressor through regulation of p21Cip1/p27Kip1 in renal cancer

**DOI:** 10.18632/oncotarget.23822

**Published:** 2017-12-26

**Authors:** Rosana D. Meyer, Xueqing Zou, Marwa Ali, Esma Ersoy, Philip Apraku Bondzie, Mehrdad Lavaei, Ilya Alexandrov, Joel Henderson, Nader Rahimi

**Affiliations:** ^1^ Department of Pathology, Boston University School of Medicine, Boston, MA 02118, USA; ^2^ Department of Hepatobiliary Surgery, Qilu Hospital of Shandong University, Jinan 250012, Shandong, China; ^3^ ActivSignal, LLC, Natick, MA 01760, USA

**Keywords:** renal cancer, tumor suppressor, TMIGD1, signal transduction, cell cycle inhibitors

## Abstract

Renal cell carcinoma (RCC) is a high-risk metastasizing tumor with a poor prognosis and poorly understood mechanism. In this study, we demonstrate that transmembrane and immunoglobulin domain-containing 1 (TMIGD1) is a novel tumor suppressor that is highly expressed in normal renal tubular epithelial cells, but it is downregulated in human renal cancer. We have identified CCAAT/enhancer-binding proteinβ (C/EBPβ, also called LAP) as a key transcriptional regulator of TMIGD1, whose loss of expression is responsible for downregulation of TMIGD1 in RCC. Transcriptionally active C/EBPβ/LAP physically interacted with and increased TMIGD1 promoter activity and expression of TMIGD1. Re-introduction of TMIGD1 into renal tumor cells significantly inhibited tumor growth and metastatic behaviors such as morphogenic branching and cell migration. Restoring TMIGD1 expression in renal tumor cells stimulated phosphorylation of p38MAK, induced expression of p21CIP1 (cyclin-dependent kinase inhibitor 1), and p27KIP1 (cyclin-dependent kinase inhibitor 1B) expression, key cell cycle inhibitor proteins involved in regulation of the cell cycle. The present study identifies TMIGD1 as a novel candidate tumor suppressor gene and provides important insight into pathobiology of RCC that could lead to a better diagnosis and possible novel therapy for RCC.

## INTRODUCTION

Kidney cancers represent about 3% of all human cancers and clear cell renal cell carcinoma (ccRCC) is the most common form of renal cancer, which is also a high-risk metastasizing tumor with a poor prognosis, and insensitive to conventional chemo/radiotherapies and targeted therapeutics [1, 2]. The biallelic functional loss of tumor suppressor gene, the von Hippel-Lindau (VHL) is the most common trademark of RCC with nearly 90% of patients carrying a somatic mutation of VHL. However, the biallelic loss of function of *VHL* is insufficient to produce ccRCC in humans and mice tumor models [3, 4], suggesting that additional genetic alterations are involved in ccRCC development.

Transmembrane and immunoglobulin domain containing (TMIGD) family proteins represent a new class of immunoglobulin (Ig) domain containing cell adhesion molecules (Ig-CAMs). The first member of the TMIGD family was identified in our laboratory as immunoglobulin and proline rich receptor-1 (IGPR-1, which is also called TMIGD2) [5]. Expression of IGPR-1 in endothelial cells regulates cell-cell adhesion, barrier function and angiogenesis [5, 6]. IGPR-1 expression in human colon cancer is increased and through promotion of multicellular aggregation it promotes tumor growth [7]. In addition to its adhesive function, IGPR-1 acts as a receptor for HERV–H LTR-associating protein 2 (HHLA2), a B7 family member, which inhibits proliferation of CD4 and CD8 T cells in the presence of T-cell receptor signaling [8, 9]. We have identified TMIGD1 as a second member of TMIGD family proteins, which is highly expressed in kidney epithelial cells and functions to inhibit kidney epithelial cell migration, and protects kidney cells from oxidative cell injury [10]. TMIGD3 represents the third member, which is reported to act as a tumor suppressor in osteosarcoma [11]. Interestingly, TMIGD3 shares its 5’ terminal exon with the adenosine A3 receptor [11]. Overall, TMIGD family proteins are composed of three major domains: extracellular, transmembrane and intracellular. The extracellular domain of TMIGD1 contains two immunoglobulin-like domains followed by a single transmembrane domain and a short intracellular domain [5, 6, 10]. The extracellular domain mediates the adhesive function of TMIGD family proteins via homophilic transdimerization [5, 6, 10].

CCAAT/enhancer-binding proteins (C/EBPs) are a family of basic leucine zipper (b-ZIP) transcription factors that bind to sequence specific double-stranded DNA to regulate gene transcription and consist of six members. C/EBPβ has three isoforms, which were originally called Liver Activating Protein 1 &2 (LAP1, LAP2) and naturally occurring transcriptionally inactive isoform called Liver Inhibiting protein (LIP) [12, 13]. C/EBPα is inactivated in multiple tumor types [12], whereas the naturally occurring dominant negatively acting C/EBPβ/LIP is upregulated in breast cancer [14], suggesting that unbalanced expression of C/EBPβ isoforms may contribute to cancer progression.

In this study, we have demonstrated that TMIGD1 acts as a tumor suppressor and its downregulation is regulated by C/EBPβ. The underlying mechanism of TMIGD1 function in inhibition of tumor growth is due its ability to modulate induction of cycle inhibitors, p21CIP1 and p27KIP1.

## RESULTS

### TMIGD1 expression is downregulated in human renal cancer

**Figure 1:**
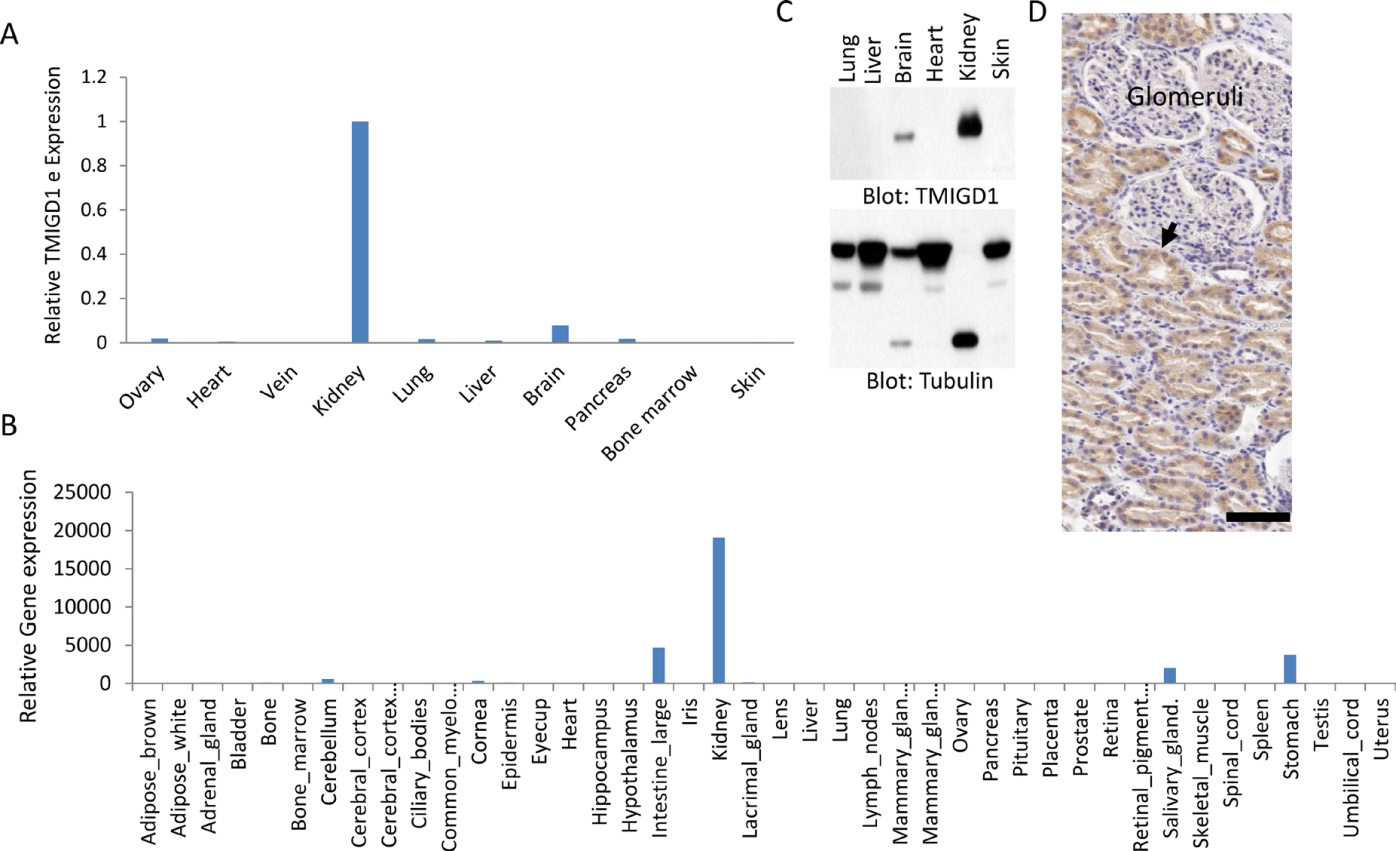
TMIGD1 expression is downregulated in human renal cancer (**A**) Total mRNA extracted from various human organs was subjected to qPCR analysis using primers specifically designed to amplify exons 2 and 3 of TMIGD1. The relative levels of IGPR-1 are shown. (**B**) TMIGD1 expression of in mouse organs/tissues is shown. The data is extracted from the online publically available mouse genome data (http://biogps.org). (**C**) Whole cell lysates from human tissues was subjected to western blot analysis using anti-TMIGD1 antibody or anti-tubulin antibody as a loading control. The lower band of tubulin is likely due to protein degerdation. (**D**) Immunohistochemistry staining of human kidney tissue using anti-TMIGD1 antibody is shown, which shows renal tubular epithelial cells are highly positive (arrow), where podocytes of the glomerulus are negative for TMIGD1. Scale bars, 50 μm.

To examine expression of TMIGD1 in human tissues and organs, we analyzed the mRNA of TMIGD1 by quantitative PCR (qPCR) using mRNA derived from a panel of human organs/tissues consisting of ovary, heart, vein, kidney, lung, liver, brain, pancreas, bone marrow and skin. The TMIGD1 mRNA was found to be highest in the kidney followed by the brain tissues. However, the TMIGD1 mRNA in the brain was significantly lower level than the kidney (Figure 1A) and its mRNA levels in ovary, heart, vein, lung, liver, pancreas, bone marrow and skin was either very low or undetectable (Figure 1A). Additionally, we analyzed the microarray data of mouse genome (http://biogps.org) for TMIGD1. Similar to human, TMIGD1 was predominately present in the mouse kidney (Figure 1B). Mouse intestine, stomach and salivary glands tissues were also positive for TMIGD1, though at the significantly lower levels (Figure 1B). Additionally, analysis of protein extract from a panel of human organs/tissues showed that TMIGD1 protein was highest in the kidney followed by brain (Figure 1C). TMIGD1 protein was not detected in lung, liver, heart or skin tissues (Figure 1C). Furthermore, immunohistochemistry (IHC) analysis of human kidney tissue demonstrated that renal tubular epithelial cells were highly positive for TMIGD1, whereas podocytes of the glomerulus were negative (Figure 1D). The specificity of anti-TMIGD1 antibody used in this study was previously validated [10]. Moreover, we have confirmed the specificity of the anti-TMIGD1 antibody in human kidney tissues in this current study. As shown, pre-incubation of the anti-TMIGD1 antibody with blocking peptide (10X) inhibited the immunoreactivity of anti-TMIGD1 antibody (Supplementary Figure 1). Taken together, the data demonstrates that TMIGD1 expression is mainly restricted to kidney epithelial cells and its expression in other human organs and tissues is either very low or it is not expressed.

To gain insight into possible function of TMIGD1 in renal cancer, we decided to examine TMIGD1 status in the publically available RCC database. Analysis of human RCC microarray data, TCGA data via online cBioPortal for Cancer Genomics (http://cbioportal.org) [15, 16], which consists of 293 cases of papillary RCC, 66 cases of chromophobe RCC and 499 cases of clear cell RCC. The TMIGD1 mRNA was downregulated in the all three major RCC types. However, downregulation of TMIGD1 in chromophobe and papillary RCC was more profound than the clear cell RCC (Supplementary Figure 2).

**Figure 2:**
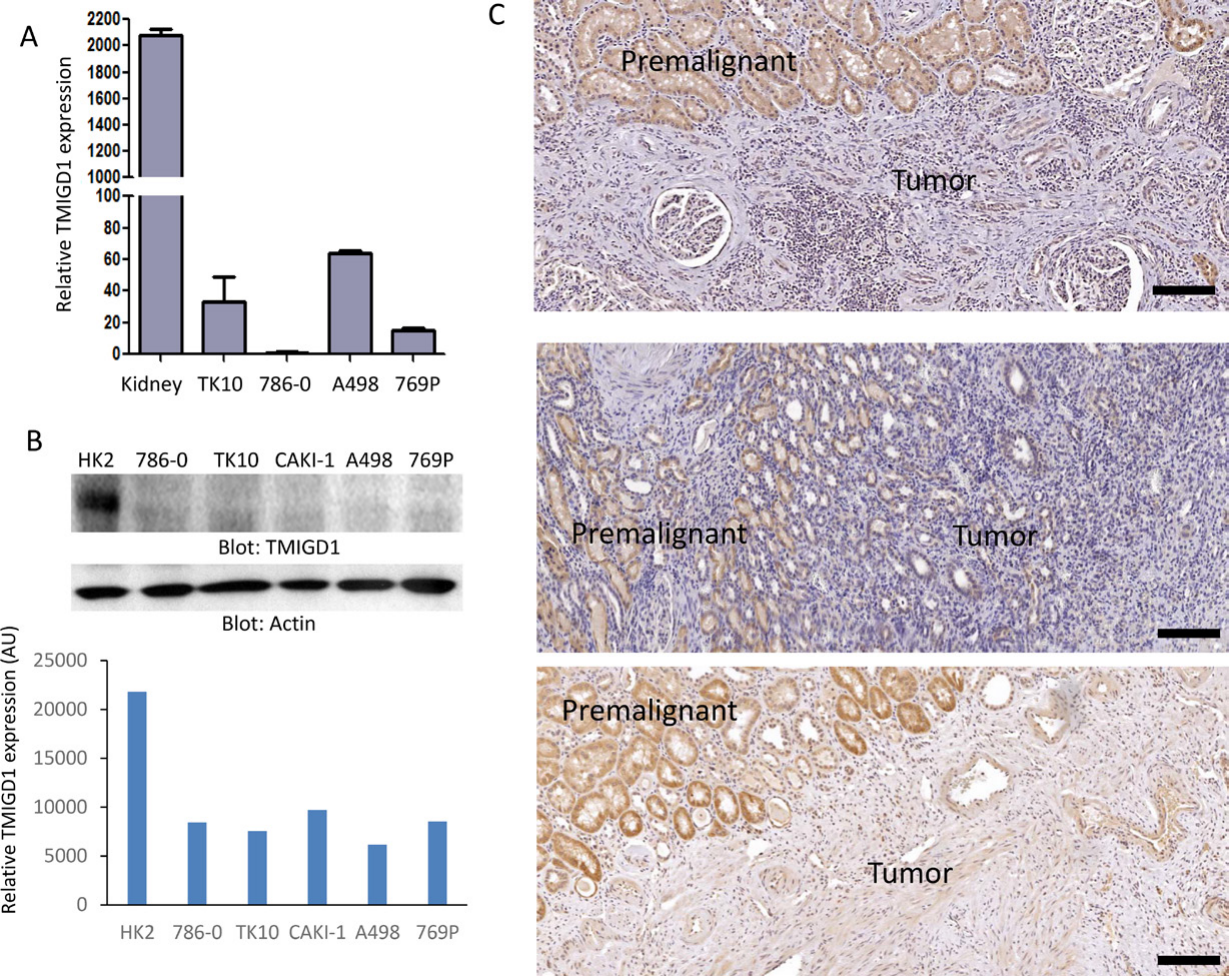
TMIGD1 expression is downregulated in human renal cancer (**A**) The mRNA of human kidney tissue or human kidney tumor cell lines including, TK10, 786-0, A498, and 769P were subjected to qPCR analysis and the relative expression of TMIGD1 is presented. (**B**) Whole cell lysates from normal human kidney cell line, HK2, or kidney tumor cell lines were blotted for TMIGD1 or for a loading control b-actin. The blot for TMIGD1 was quantified using the Image J software. AU (arbitrary unit). (**C**) Human kidney cancer tissues were subjected to immunohistochemistry staining using anti-TMIGD1 antibody and three representative images were shown. Scale bars, 50 μm.

These observations encouraged us to examine TMIGD1 expression in human RCC cell lines and tissues. Our analysis showed that TMIGD1 mRNA was considerably lower in renal tumor cell lines including, 786-0, A498, 769P and TK10 compared to normal kidney mRNA (Figure 2A). Likewise, while TMIGD1 protein was present in the normal human kidney epithelial cell line, HK2, its expression was hardly detectable in 786-0, TK10, CAKI-1, A498, and 769P cell lines (Figure 2B). A longer exposure of the film detected a faint protein band (data not shown).

Next, we examined a cohort of 24 human renal cancer biopsy samples for expression of TMIGD1. The cohort consisted of 13 cases of renal cell carcinomas (RCC), and 11 cases of benign kidney lesions or normal kidney tissues (Figure 2C). The IHC analysis revealed a striking downregulation of TMIGD1 in RCC compared to normal adjacent tissue or the other normal/benign kidney tissues (Figure 2C). Expression of TMIGD1 was unchanged in the non-cancerous renal disease, nephrosclerosis (3 cases), compared to the normal/non-diseased kidney tissues (data not shown). Altogether, the data demonstrates that TMIGD1 in the RCC cell lines and in primary RCC tumors is significantly downregulated, suggesting that the reduced expression of TMIGD1 in RCC could play a role in tumor progression.

### TMIGD1 expression in renal tumors inhibits tumor growth and cell migration

**Figure 3:**
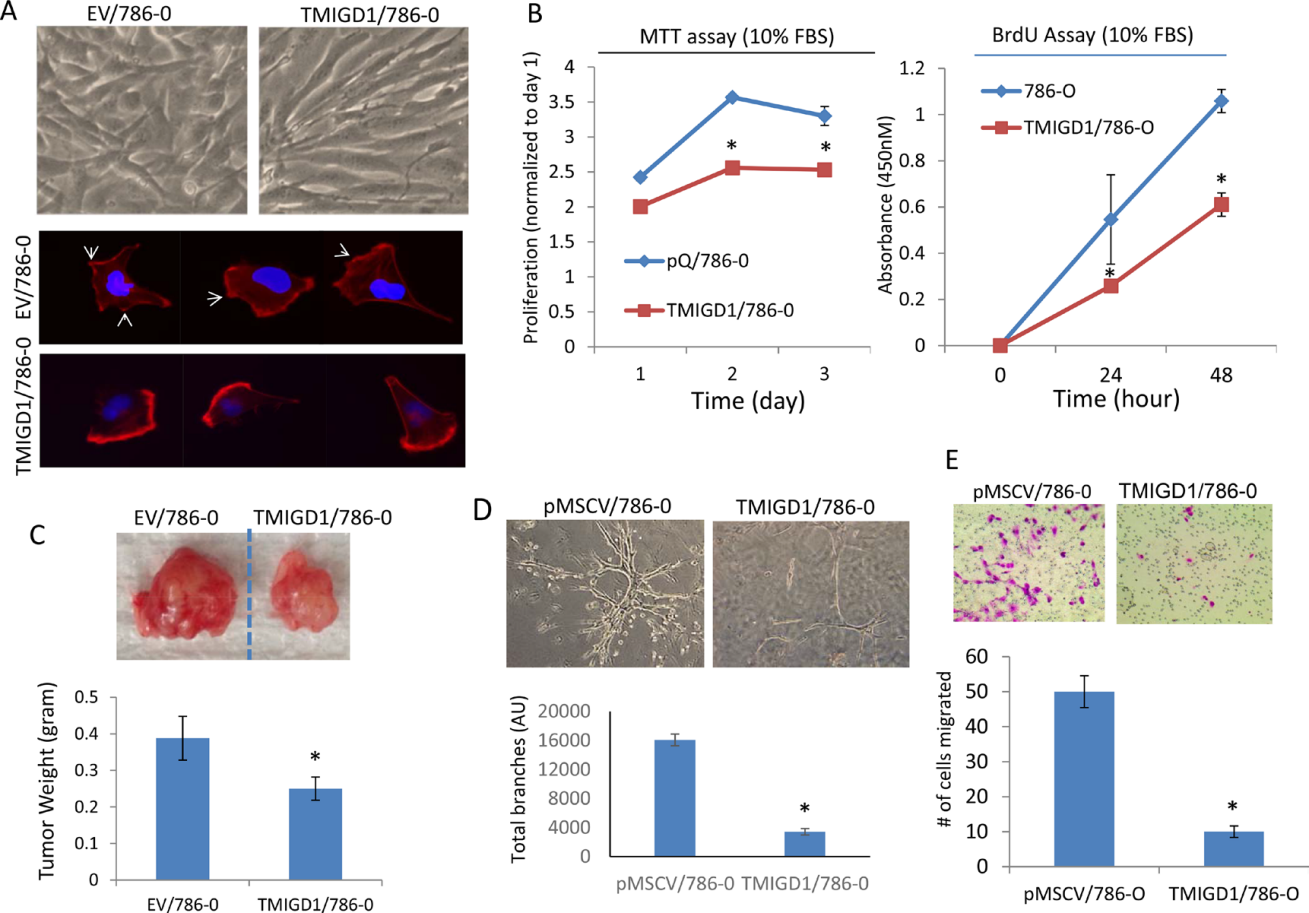
Re-expression of TMIGD1 in 786-0 cells inhibits tumor growth, cell invasion and branching morphogenesis (**A**) Morphology of 786-0 cells expressing empty vector (EV) or TMIGD1 seeded on the collagen-coated plates for 24 hours. The same cells were seeded on the collagen-coated cover slips for 40 minutes, cells were fixed and stained with Rhodamine Phalloidin and pictures (40X) were taken under an immunofluorescence microscope. (B) Equal number of 786-0 cells expressing empty vector (EV) or TMIGD1 were plated in 24-well plates in quadruple in the presence of 10%FBS and proliferation of cells were measured at day 1, 2 and 3. ^*^p < 0.05. (**B**) Similarly, equal number of 786-0 cells expressing empty vector or TMIGD1 were plated in 24-well plates in quadruple and subjected to BrdU staining and cell proliferation was measured at day 0, 24 h and 48 h. Experiments are presentative of three intendent experiments. ^*^p < 0.05. (**C**) Equal number of 786-0 cells expressing empty vector (pMSCV) or TMIGD1 were mixed with matrigel and xenografted into athymic nude mice (4/group) and after 27 days tumors were excised, weighed and pictures were taken. *P* = 0.033. (**D**) 786-0 cells expressing empty vector or TMIGD1 were subjected to branching morphogenesis and pictures were taken after 9 days. Branching morphogenesis (four images/group) was quantified via Image J software. ^*^p < 0.05. The data is representative of three independent experiments. (**E**) 786-0 cells expressing empty vector or TMIGD1 were seeded on the collagen-coated transwells in triplicates for about 12 hours. Cells were fixed and number of cells that were migrated to the other side of the transwells were counted under microscope. The data is representative of three independent experiments (quadruple wells/group). ^*^p < 0.05.

Considering the stark downregulation of TMIGD1 in human RCC tumors, we hypothesized that downregulation of TMIGD1 in renal tumors could play an important role in the biology of renal tumor cells. Therefore, we re-introduced TMIGD1 into a renal tumor cell line, 786-0 cells via a retroviral system and assessed its function in proliferation of 786-0 cells. Ectopic expression of TMIGD1 in 786-0 cells is shown (Supplementary Figure 3). Additionally, expression of TMIGD1 in 786-0 cells also altered the morphology of these cells (Figure 3A). Rhodamine-phalloidin staining for actin showed that 786-0 cells expressing TMIGD1 seeded on the collagen-coated plate showed that in TMIGD1 expressing 786-0 cells, the actin fibrils were distinctively enriched in an asymmetrical fashion. These cells appeared to show no clear filopodia/lamellipodia-like protrusive structures compared to the control 786-0 cells expressing an empty vector (EV) (Figure 3A). Taken together, the data suggest that TMIGD1 ectopically expressed in 786-0 cells promotes morphological changes, which may account for potential role of TMIGD1 in cell differentiation.

Next, we measured proliferation of 786-0 cells expressing TMIGD1 or empty vector via MTT ((3-(4,5-dimethylthiazol-2-yl)-2,5-diphenyltetrazolium bromide) and BrdU (5-bromo-2’-deoxyuridine) assays. The results showed that expression of TMIGD1 in 786-0 cells significantly inhibited cell proliferation compared to 786-0 cells expressing an empty vector (Figure 3B). Having observed the inhibitory effect of TMIGD1 in 786-0 cells, we examined the effect of TMIGD1 expression in the tumor formation of 786-0 cells in an athymic nude mouse. Consistent with its effect in cell culture, 786-0 cells expressing TMIGD1 formed significantly smaller tumors compared to 786-0 cells expressing empty vector in mouse (Figure 3C).

Having confirmed the ability of TMIGD1 to inhibit tumor growth in cell culture and in mouse, we sought to investigate the possible role of TMIGD1 in the invasive characteristics of 786-0 cells. Thus, we subjected 786-0 cells expressing empty vector or TMIGD1 to an *in vitro* 3D branching morphogenesis assay. Branching morphogenesis is an essential developmental process that involves the restructuring of epithelial tissues into organized ramified tubular network and also plays an important role in tumor cell invasion and metastasis [17]. Our data showed that 786-0 cells expressing empty vector grown in collagen gel exhibited extensive branching morphogenesis and invasiveness (Figure 3D). In contrast, 786-0 cells expressing TMIGD1 displayed only a few discrete branching morphogenesis (Figure 3D), indicating that TMIGD1 inhibited the invasive potential of 786-0 cells. A similar effect observed when TMIGD1 was expressed in A498 cells (data not shown). Next, we examine the possible role of TMIGD1 in cell migration and show that TMIGD1 inhibited migration of 786-0 cells (Figure 3E). Altogether, the data demonstrates that re-expression of TMIGD1 in 786-0 cells inhibits tumor growth and invasion. Hence, the data also suggests that downregulation of TMIGD1 in renal tumor cells is associated with increased tumor growth and invasion.

### TMIGD1 induces expression of cell cycle inhibitors, p21CIP1 and p27KIP1 to inhibit tumor growth

**Figure 4:**
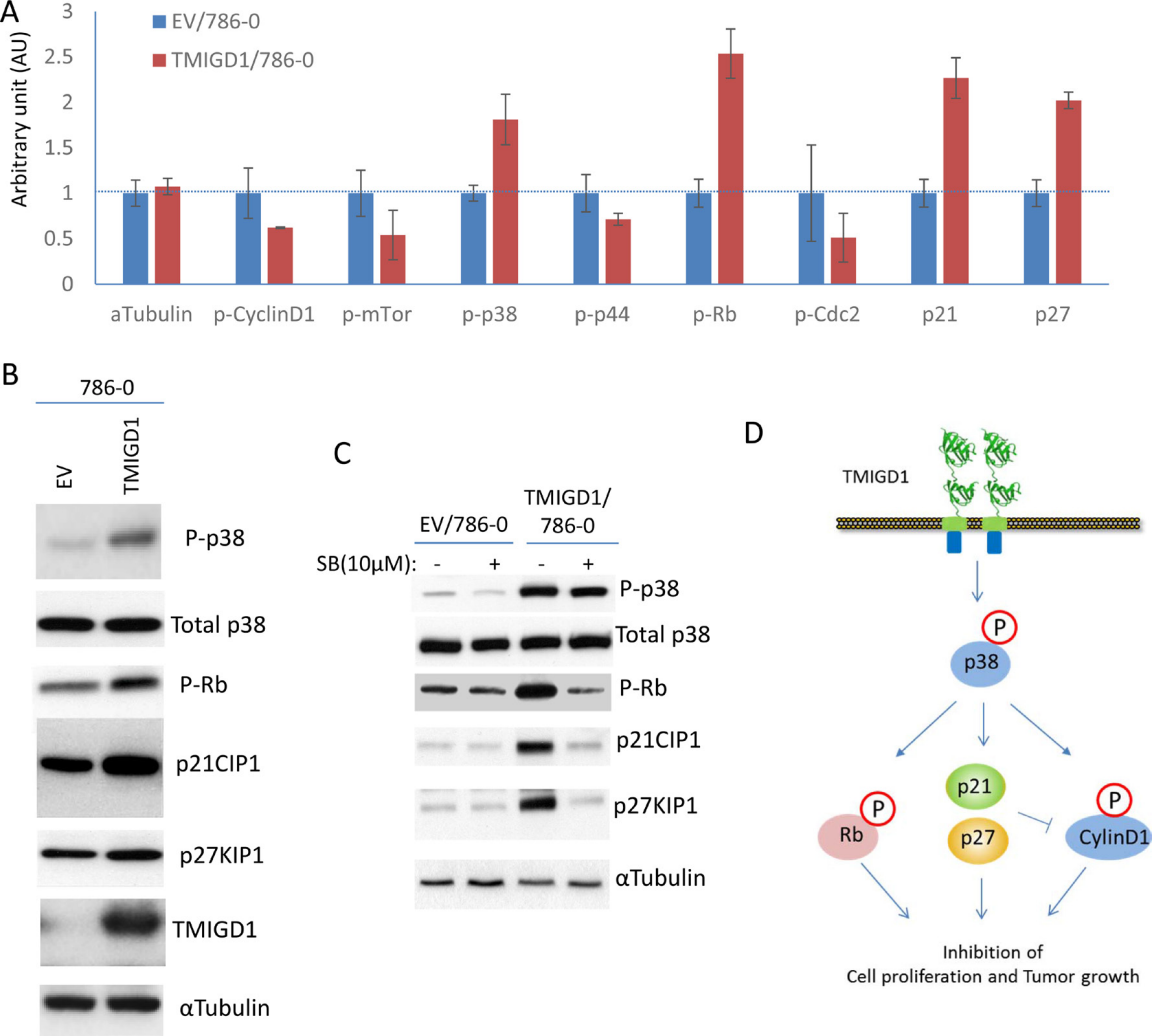
Re-expression of TMIGD1 in 786-0 cells induces multiple pathways involved in the inhibition of cell cycle and cell proliferation (**A**) 786-0 cells expressing empty vector (EV) or TMIGD1 were plated in 96-well plates in triplicates and subjected to ActiveSignal assay Assay analysis, which measures expression or activation of 70 proteins . The graph shows the major proteins involved in the regulation of cell cycle and are affected by TMIGD1. AU (arbitrary unit). (**B**) Whole cell lysates from 786-0 cells expressing empty vector (EV) or TMIGD1 were subjected to western blot analysis and western blots of a panel of selected proteins involved in the regulation of cell cycle and proliferation are shown. (**C**) 788-0 cells expressing empty vector or TMIGD1 were treated with p38-MAPK inhibitor (SB203580) for 30 minutes. Cells were lysed and whole cell lysates were blotted for total p38, phospho-p38, phospho-Rb, p21CIP1 and p27KIP1. (**D**) Schematic of pathways affected by TMIGD1 in 786-0 cells and the possible role of p38MAPK in the regulation of phospho-Rb, and induction of p21CIP1 and p27KIP1.

How does TMIGD1 stimulate anti-proliferative responses in RCC tumor cells? To address this question, we analyzed 786-0 cells expressing TMIGD1 for activation of twenty major cancer pathways consisting of 70 individual proteins via a recently developed immuno-paired-antibody detection system (ActiveSignal Assay) analysis platform. Among the major pathways that were affected by TMIGD1 in 786-0 cells were the proteins that are known to inhibit cell cycle and cell proliferation. Specifically, TMIGD1 upregulated expressions of p21CIP1 (cyclin-dependent kinase inhibitor 1) and p27KIP1 (cyclin-dependent kinase inhibitor 1B expression), proteins whose expression are critically important for negative regulation of the G1-phase cell cycle progression [18]. Additionally, while TMIGD1 reduced phosphorylation of CyclinD1 and Cyclin-dependent kinase 1 (CDK1/Cdc2), it increased phosphorylation of retinoblastoma protein (Rb), and p38MAPK (MAPK14) (Figure 4A). The effect of TMIGD1 on the expression or phosphorylation of these proteins was confirmed further by western blot analysis (Figure 4B). Based on these observations, we propose that phosphorylation of p38 by TMIGD1 plays a central role in the TMIGD1's anti-proliferative function in RCC tumor cells. Activation of p38 by TMIGD1 could phosphorylates CyclinD1 and RB and induction of p21CIP1 and p27KIP1 as treatment of cells with p38 inhibitor, SB203580 inhibited phosphorylation of Rb and induction of p21CIP1 and p27KIP1 (Figure 4C). p38 kinase is considered a tumor suppressor whose function can negatively regulate cell cycle progression and apoptosis [19–21]. Activation of p38 has been shown to induce cell cycle arrest by promoting expression or stabilization of p21CIP1 [22–25] and p27KIP1 ([26, 27] by various mechanisms. Moreover, p38 kinase can phosphorylate Cyclin D1, which leads to its degradation [28, 29]. Cyclin D1 is a proto-oncogene that is upregulated in various cancers and plays an important role in transition of G1 to S phase progression by binding to cyclin-dependent kinases such as CKD2 and CDK4 that promotes cell cycle through inhibition of phosphorylation of retinoblastoma protein (Rb). Activated p38 also can phosphorylation Rb [30, 31], which leads to its degradation. A recent study demonstrates that Phosphorylation of RB by p38 bypasses its inactivation by CDKs and inhibits proliferation of cancer cells [31].

Taken together, our data demonstrates that restoring expression of TMIGD1 in 786-0 cells stimulates p38 activation, induces p21CIP1 and p27KIP1, and inhibits tumor growth in cell culture and in athymic mouse. These findings also point to TMIGD1 as a novel protein whose downregulation in renal cancer could be associated with tumor malignancy.

### CCAAT/enhancer-binding protein (C/EBP-β) regulates promoter activity of TMIGD1

**Figure 5:**
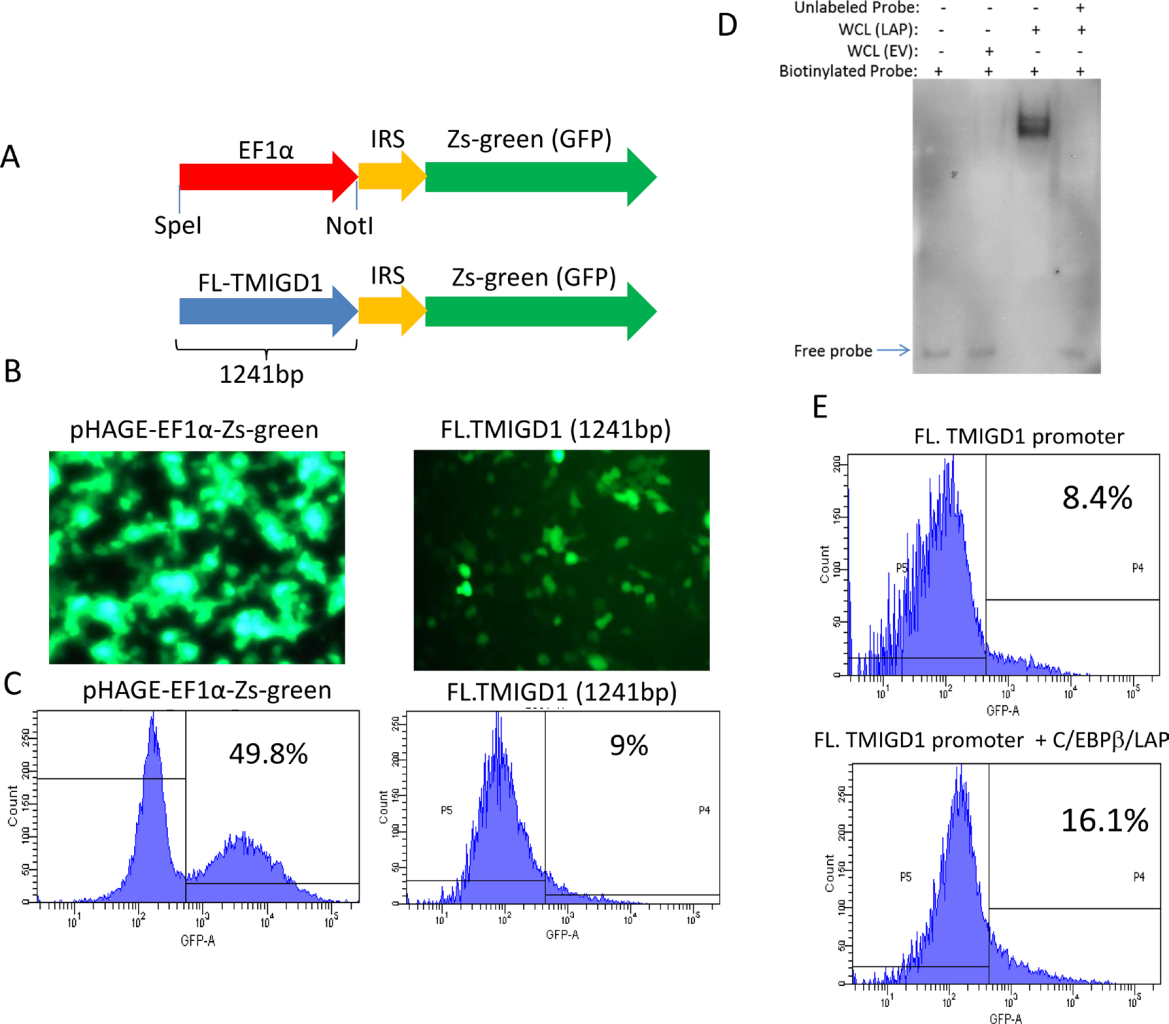
C/EBPβ transcription factor regulates expression of TMIGD1 (**A**) Shown is the schematic of full-length TMIGD1 promoter cloned into pHAGE-GFP reporter vector. (**B**) HEK-293T cells were transfected with TMIGD1- pHAGE-GFP or EFα1-pHAGE-GFP. Images were taken under a fluorescent microscope (20X) after 48 hours of transfection. (**C**) TMIGD1 and control EFα1 promoter activities was quantified by FACS analysis via measuring expression of GFP. (**D**) 5’ biotinylated TMIGD1 promoter mixed either with the cell lysates of HEK-293 cells over-expressing C/EBPβ (LAP) or empty vector and subjected to an electrophoretic mobility shift (EMSA) assay. (**E**) HEK-293T cells were transfected with full-length (FL) TMIGD1 promoter alone or with C/EBPβ (LAP). After 4 days transfection, cells were prepared and subjected to FACS analysis. The data presented in this figure were independently repeated at least three times.

To gain further insights into a possible mechanism of downregulation of TMIGD1 in RCC, we cloned the 5’-flanking non-coding region of TMIGD1 encompassing 1,241 base pairs (bp), located upstream of the start site of transcription in the TMIGD1 gene. We subsequently cloned the TMIGD1 promoter into a GFP (Zgreen) reporter vector and analyzed its promoter activity by monitoring the expression of GFP (Figure 5A). The full-length (1,241bp) TMIGD1 promoter and control elongation factor-1α (EF1α) promoter were transfected into HEK-293T cells. HEK-293 cells transfected with full-length TMIGD1 promoter showed a moderate transcription activity (9%) compared to EF1α (49.8%) (Figure 5B, 5C).

Next, we sought to identify possible transcription factors involved in the regulation of transcription of TMIGD1. We used LASAGNA-Search 2.0, an integrated web tool for transcription factor binding site search and visualization (http://biogrid-lasagna.engr.uconn.edu/lasagna search/ ) [32]. We considered CCAAT/enhancer-binding protein (C/EBP) as a potential transcription factor involved in the regulation of TMIGD1 as multiple C/EBP binding sites (TTGCnnAA ) were predicated on the TMIGD1 promoter.

CCAAT/enhancer-binding protein (C/EBP) family members are structurally highly conserved and are member of the basic leucine zipper (bZIP) transcription factors, which bind selectively to CCAAT box sequences with the GGCCAATCT consensus and can function as both tumor promoters and tumor suppressors [12, 33]. Currently, there are six known C/EBP proteins; α, β, γ, δ, ε and CHOP10 [34, 35]. Among the C/EBPs, C/EBPα and C/EBPβ isoforms are the most widely expressed, and most well studied [12]. C/EBPβ functions as a homodimer but can also form heterodimers with other C/EBP family proteins [12]. To examine the possible role of C/EBPβ in the transcriptional regulation of TMIGD1, we first asked whether C/EBPβ physically interacts with the TMIGD1 promoter. To this end, we examined the binding of C/EBPβ with TMIGD1 via electrophoretic mobility shift assay (EMSA) assay and demonstrated that C/EBPβ strongly interacts with the TMIGD1 promoter (Figure 5D). The finding identifies C/EBPβ as a possible transcriptional regulator of TMIGD1. To investigate the potential functional role of C/EBPβ in the transcriptional regulation of TMIGD1, we co-expressed C/EBPβ (LAP, transcriptionally active form of C/EBPβ) with the full-length TMIGD1 promoter in HEK-293T cells. C/EBPβ (LAP) increased the promoter activity of TMIGD1 nearly by 90% (Figure 5E). Expression of C/EBPβ (LAP) is shown (Supplementary Figure 4A). In contrast to the stimulatory effect of C/EBPβ (LAP) on the promoter activity of TMIGD1, over-expression of a naturally occurring transcriptionally inactive C/EBPβ (LIP), inhibited the TMIGD1 promoter activity Supplementary Figure 6D. For this particular experiment we used a truncated TMIGD1 promoter, which 646 bp from the 5′ flanking via naturally occurring *speI* restriction site (–1241 to –595) of TMIGD1 promoter was deleted. We used the truncated TMIGD1 promoter because it has better promoter activity than the full-length TMIGD1 promoter, therefore the potential repressor effect of C/EBPβ (LIP), could be demonstrated better. C/EBPβ (LIP) inhibited the promoter activity of TMIGD1 (Supplementary Figure 6B). Expression of C/EBPβ (LIP) also is shown (Supplementary Figure 6C). Collectively, the data demonstrates that C/EBPβ binds to, and regulates the promoter activity of TMIGD1.

### C/EBPβ expression is downregulated in human renal cancer and its re-expression promotes expression of TMIGD1

**Figure 6:**
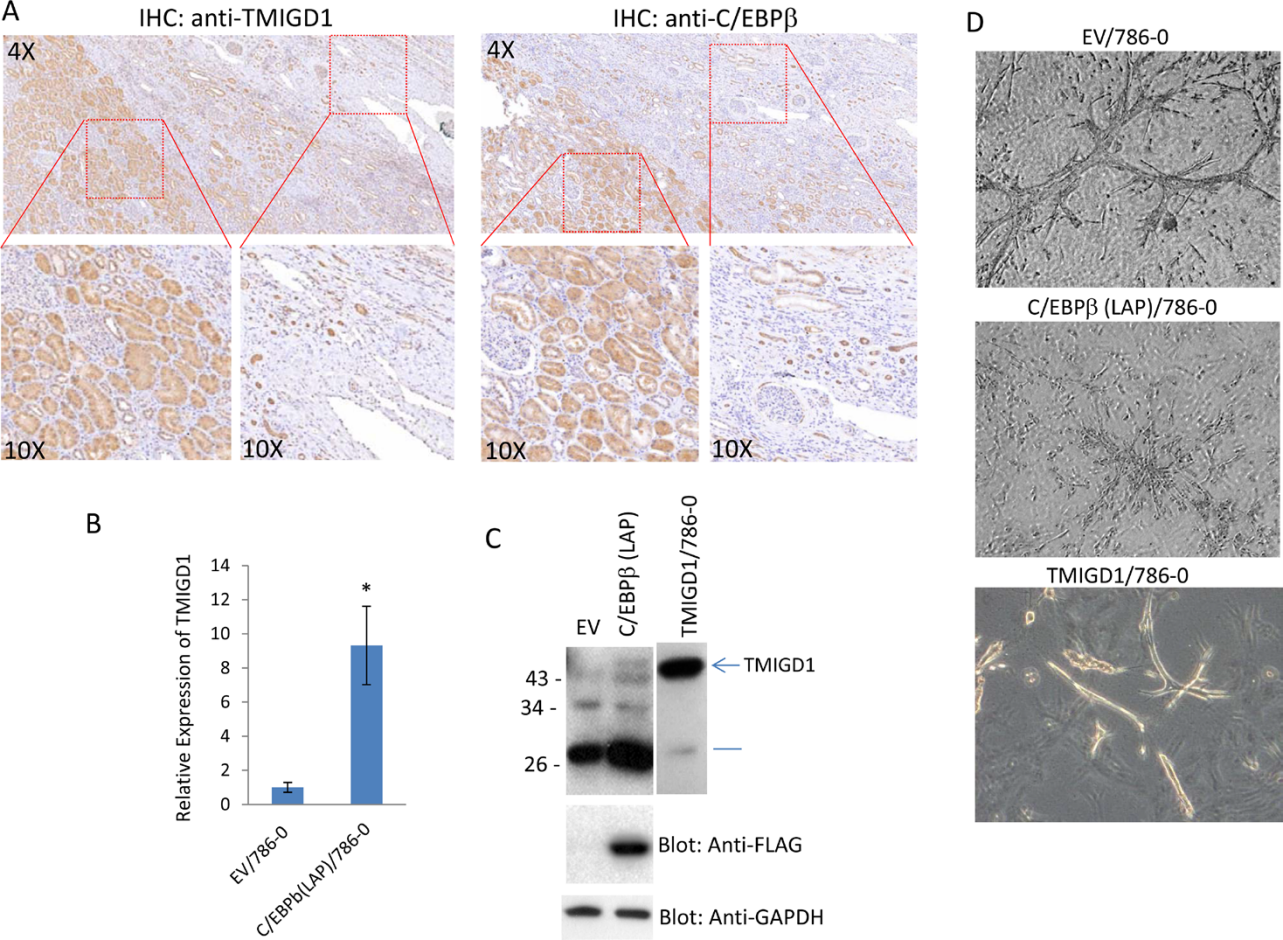
C/EBPβ regulates expression of TMIGD1 in renal tumor (**A**) Human kidney cancer tissues (5 cases) were subjected to IHC staining using anti-TMIGD1 antibody or anti-C/EBPβ antibody and representative images are shown. (**B**) 786-0 cells were transfected with either an empty vector or C/EBPβ (LAP) and the mRNA derived from cells subjected to qPCR analysis. GAPDH was used for internal control. *P* = 0.0058. (**C**) Similarly, 786-0 cells expressing empty vector or C/EBPβ (LAP) were lysed, subjected to western blot analysis and blotted for TMIGD1, C/EBPβ (LAP) using anti-FLAG antibody and GAPDH for loading control. (**D**) 786-0 cells 786-0 cells expressing empty vector or C/EBPβ (LAP) were prepared and subjected to branching morphogenesis assay. Pictures were taken under a microscope after 10days.

We posited that downregulation of TMIGD1 in renal tumor could be associated with the altered expression of C/EBPβ. We first examined expression of C/EBPβ in human renal cancer cell lines. Expression of C/EBPβ (LAP) in 786-0, DLD1 and TK10 was very low or undetectable. However, C/EBPβ (LAP) was readily detected in normal human mouse renal epithelial cells (Supplementary Figure 5). Next, we examined C/EBPβ and TMIGD1 expressions in the human RCC tumor tissues. The result showed that expression of C/EBPβ in renal cancer is significantly reduced (Figure 6A) and its reduced expression closely correlated with the expression of TMIGD1 (5 out 5 cases) (Figure 6A). Expression of both TMIGD1 and C/EBPβ in normal adjacent renal tissue were high, where expressions of both were significantly low in the tumor region (Figure 6A). The data suggests that reduced expression of C/EBPβ could in part explain the downregulation of TMIGD1 in renal tumors. To test this possibility, we over-expressed C/EBPβ in 786-0 cells and examined expression of TMIGD1 both at the mRNA and protein levels. Remarkably, expression of C/EBPβ (LAP) in 786-0 cells, increased expression of TMIGD1 mRNA and protein (Figure 6B and 6C). Analysis of cell lysates from 786-0 cells that expressed C/EBPβ (LAP)-FLAG showed an increase in TMIGD1 protein at the about 45kDa, which corresponds to mature (fully glycosylated) TMIGD1 protein (Figure 6C). In addition, C/EBPβ (LAP)-FLAG expression in 786-0 cells increased expression of a low molecular weight TMIGD1 protein with an approximate molecular weight of 29kDa (Figure 6C). The predicted molecular weight of TMIGD1 is 29 kDa [10], suggesting that the lower molecular weight band likely corresponds to unprocessed (non-glycosylated) form of TMIGD1. Moreover, we asked whether overexpression of C/EBPβ (LAP)-FLAG in 786-0 cells could inhibit branching morphogenesis. The result showed that 786-0 cells expressing C/EBPβ (LAP)-FLAG displayed a significantly reduced branching morphogenesis (Figure 6D), further supporting a relationship between expression of C/EBPβ and TMIGD1, in which downregulation of C/EBPβ leads to suppression of TMIGD1 expression.

## DISCUSSION

The present study has uncovered a previously unknown function forTMIGD1 in renal cancer. TMIGD1 is downregulated in human RCC tumors and RCC tumor cell lines. We have demonstrated the mechanism by which expression of TMIGD1 in renal tumors is regulated by transcriptional activity of C/EBPβ. While expression of both TMIGD1 and C/EBPβ are relatively high in normal renal epithelia, expression of both are significantly downregulated in RCC. C/EBP transcription factors could act as tumor suppressor or tumor promoter [36–38]. C/EBPα is inactivated in multiple tumor types [12], whereas the naturally occurring dominant negatively acting C/EBPβ (LIP) is upregulated in breast cancer, suggesting that unbalanced expression of C/EBPβ isoforms may contribute to cancer progression [14]. Our data favors a tumor suppressor function for C/EBPβ in renal tumor and similar to breast cancer altered expression of transcriptionally inactive C/EBPβ (LIP) or suppression of expression of transcriptionally active C/EBPβ (LAP) could in part contribute to renal tumor progression by modulating target genes such as TMIGD1. Multiple recent studies have shown that expression of C/EBPα in tumor cells induces cell cycle arrest [35, 39, 40]. Similarly, expression of C/EBPβ in various tumor types induced cell cycle arrest [35, 41, 42].

More importantly, the data presented in this present work suggests that reduced expression of TMIGD1 by renal tumor cells provide a significant gain for their tumorigenic properties. Based on these observations we propose TMIGD1 as a candidate tumor suppressor gene. In agreement with our view of TMIGD1 as a candidate tumor suppressor gene, a recent study demonstrated that TMIGD1 expression was also downregulated in human pre-invasive colorectal cancer [43]. Additionally, a TMIGD1 related protein, TMIGD3, was recently described as a tumor suppressor in osteosarcoma [11]

**Figure 7:**
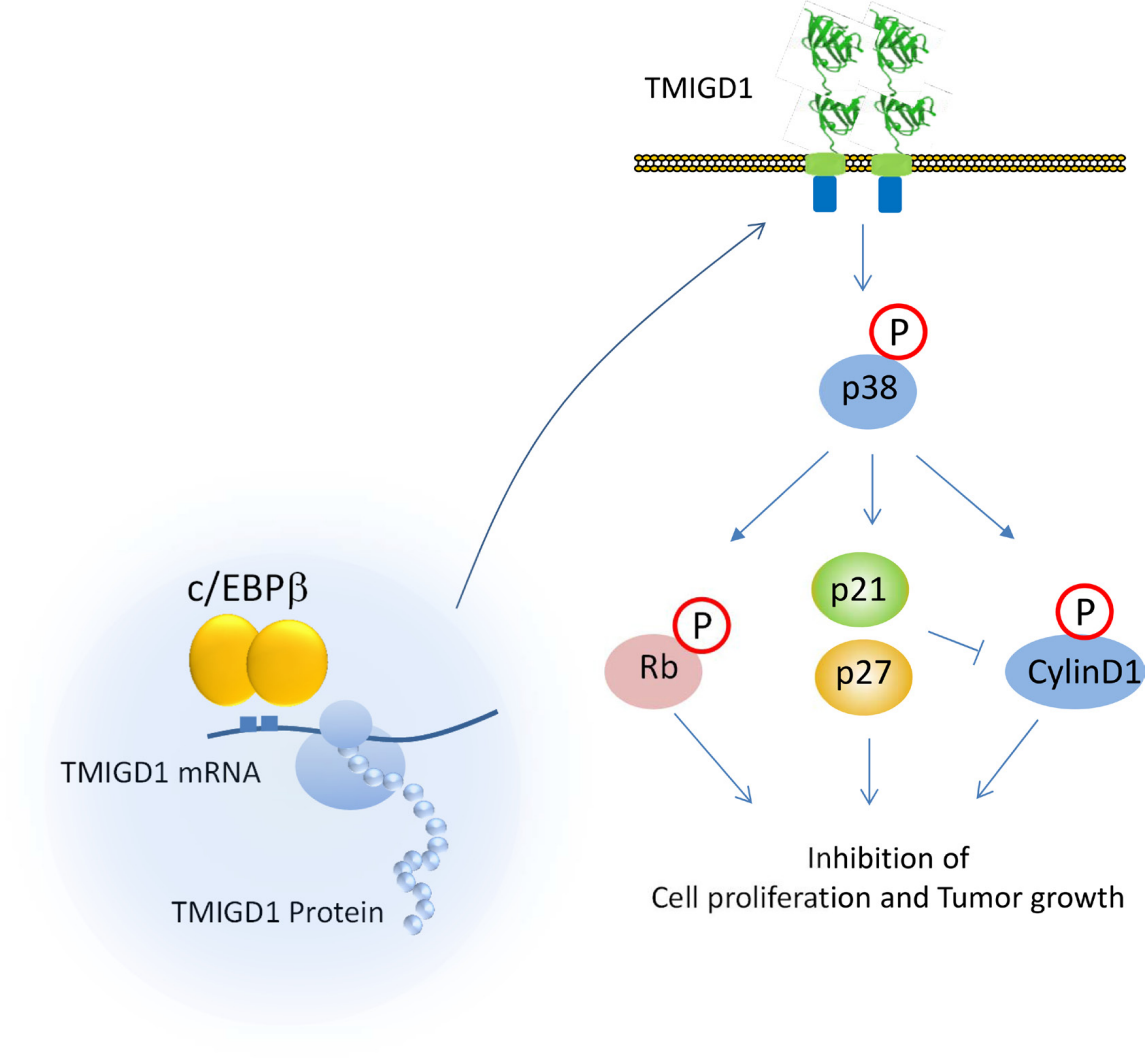
Proposed model for expression and signaling of TMIGD1 Based on the data presented in this study, we propose that downregulation of C/EBPβ or its activity in renal cancer cells is associated with suppression of TMIGD1 expression in RCC. Restoring TMIGD1 expression in renal tumor cells activated p38-MAPK that lead to induction of p21CIP1/p27KIP1 that inhibits cell proliferation and migration.

Re-introduction of TMIGD1 into 786-0 cells affected expression or function of several key signaling proteins involved in the regulation of cell cycle and cell proliferation. In particular, TMIGD1 upregulated expressions of p21CIP1 and p27KIP1, and increased phosphorylation of Rb, which are considered the master regulators of cell cycle and cell proliferation. p21CIP1 and p27KIP1 are atypical tumor suppressors that regulate G0 to S phase transitions by binding to and regulating the activity of cyclin-dependent kinases (Cdks) [44, 45]. Expression of p27KIP1 and p21CIP1 are often induced by cell-cell contact dependent fashion to inhibit cyclin-D and Cdks and hence facilitate the inhibition of cell proliferation [46, 47]. We propose that TMIGD1 modulates these key pathways involved in the regulation of cell cycle by promoting activity of p38, as activation of p38 is known to regulates expression of p21CIP1, p27KIP1, phosphorylation of cyclin D1 and Rb [22–27] (Figure 7). Cyclin D1 is involved in the transition of G1 to S phase progression by binding to cyclin-dependent kinases that promotes cell cycle through inhibition of phosphorylation of Rb. Phosphorylation Cyclin D1 and Rb by p38 kinase leads to their degradation of Cyclin D1 [28–31].

Our findings have important implications in the diagnosis and possible management of renal cancer. Targeting the C/EBPβ /TMIGD1 pathway that can lead to restoration of TMIGD1 expression offers a novel therapeutic strategy to treat RCC. However, further studies will be required to establish the molecular mechanism of this novel pathway in kidney cancer progression.

## MATERIALS AND METHODS

### Cell culture and cell lines

Human renal cell carcinomas, 786-0, TK10, A498, and 769P were kindly provided by Dr. Herbert Cohen, Boston University Medical Campus and maintained in RPMI plus 10% fetal bovine serum (FBS) and Penicillin/Streptomycin. 786-0 cells expressing TMIGD1 were established via a retroviral system as previously described [10]. Retroviruses were produced in 293-GPG cells [48]. Viral supernatants were collected for 3 days, concentrated viruses were used to transduce into 786-0 cells, and infected cells were selected with puromycin.

### Antibodies, plasmids and primers

Rabbit polyclonal anti-TMIGD1 antibody was made against the extracellular domain of TMIGD1 as described [10]. The list of plasmids, primers and other antibodies that were used in this study are described in the supplementary document.

### Branching-morphogenesis assay

786-0 cells expressing either empty vector or TMIGD1 were subjected to branching-morphogenesis assay as described with some modifications [49]. Full description of the assay is provided in the supplementary data document.

### Immunohistochemistry analysis

RCC biopsies of patients (24 cases) were obtained from the Department of Pathology, Boston University School of Medicine. Immunohistochemistry (IHC) staining was performed as per the manufacturer’s instruction using the EXPOSE Rabbit specific HRP/DAB detection IHC kit (Abcam, Cambridge, USA). Slides were scanned via iScan Coreo Au scanner (Ventana, AZ, USA). Two surgical pathologists independently evaluated the staining of TMIGD1 and C/EBPβ.

### Cell spreading assay

Cells were plated on collagen-coated cover slips and incubated at 37°C incubator for 40 minutes. Cell were fixed with 4%PFA for 15 minutes and stained with Rhodamine Phalloidin for actin (Molecular Probes cat#R415) as described [5] and pictures were taken using an immunofluorescence microscope.

### Cell proliferation assays

The 3-(4,5-dimethylthiazol-2-yl)-2,5-diphenyltetrazolium bromide (MTT) cell proliferation assay was performed as described by the manufacturer (Promega LLC, Madison, USA) and read at an absorbance of 570 nm in microplate reader (VERSA max, Molecular Devices, Sunnyvale, CA). BrdU (5-bromo-2’-deoxyuridine) assay was also used to measure cell proliferation and performed as described by the manufacturer (EMD Millipore, Billerica, USA). Cell proliferation assay was performed in 24-well plates in quadruple and repeated three times.

### Athymic mouse tumor xenograft assay

Female NUD mice (5–6 weeks old, 5 mice/group) were obtained from Jackson Laboratories. Each mouse was injected subcutaneously in the right flank with 786-0 cells expressing empty vector or TMIGD1 (5 × 10^6^) mixed with growth factor-reduced and phenol red free Matrigel (Corning, Inc.). Tumor xenografts were grown for 25 days before animals were sacrificed and xenografts were removed, photographed and measured.

### Immuno-paired-antibody detection (ActivSignal assay) analysis

ActivSignal assay examines phosphorylation or expression of 70 different human protein targets, which covers 20 major signaling pathways. ActiveSignal assay uses paired antibodies for each target protein and detection occurs only if both antibodies in a pair bind to a specific target protein. The detection of the paired antibodies is facilitated via a special DNA barcodes conjugated to antibodies, which quantified using Next Generation Sequencing or the Fluidigm digital PCR platform.

### Promoter activity assay/FACS analysis

HEK-293T cells were transfected in triplicates with the full-length TMIGD1 or truncated TMIGD1 promoters alone or together with C/EBPβ (LAP) or with C/EBPβ (LIP) constructs. After about 96 hours of transfection, cells were trypsinized, suspended in PBS and subjected to FACS analysis (Boston University Flow cytometry core facility).

### Electrophoretic mobility shift assay (EMSA) assay

Cell lysate from HEK-293T cells expressing C/EBPβ (LAP)-FLAG was used as a source of C/EBPβ. Biotin-labeled TMIGD1 promotor probe (-595 to -1) was generated by PCR using 5’ biotinylated primer. The detail of the procedure is described in the supplementary document.

### Statistical analyses

All data in the text and figures are provided as means ± SD. The Student’s two-tailed *t*-test (assuming equal variances) was used to analyze the cell proliferation data in experiments comparing two cell lines. A *p*-value of less than 0.05 was considered statistically significant.

## SUPPLEMENTARY MATERIALS FIGURES



## References

[1] Linehan WM, Srinivasan R, Schmidt LS (2010). The genetic basis of kidney cancer: a metabolic disease. Nat Rev Urol.

[2] Pirrotta MT, Bernardeschi P, Fiorentini G (2011). Targeted-therapy in advanced renal cell carcinoma. Curr Med Chem.

[3] Mandriota SJ, Turner KJ, Davies DR, Murray PG, Morgan NV, Sowter HM, Wykoff CC, Maher ER, Harris AL, Ratcliffe PJ, Maxwell PH (2002). HIF activation identifies early lesions in VHL kidneys: evidence for site-specific tumor suppressor function in the nephron. Cancer Cell.

[4] Frew IJ, Moch H (2015). A clearer view of the molecular complexity of clear cell renal cell carcinoma. Annu Rev Pathol.

[5] Rahimi N, Rezazadeh K, Mahoney JE, Hartsough E, Meyer RD (2012). Identification of IGPR-1 as a novel adhesion molecule involved in angiogenesis. Mol Biol Cell.

[6] Wang YH, Meyer RD, Bondzie PA, Jiang Y, Rahimi I, Rezazadeh K, Mehta M, Laver NM, Costello CE, Rahimi N (2016). IGPR-1 Is Required for Endothelial Cell-Cell Adhesion and Barrier Function. J Mol Biol.

[7] Woolf N, Pearson BE, Bondzie PA, Meyer RD, Lavaei M, Belkina AC, Chitalia V, Rahimi N (2017). Targeting tumor multicellular aggregation through IGPR-1 inhibits colon cancer growth and improves chemotherapy. Oncogenesis.

[8] Janakiram M, Chinai JM, Zhao A, Sparano JA, Zang X (2015). HHLA2 and TMIGD2: new immunotherapeutic targets of the B7 and CD28 families. OncoImmunology.

[9] Zhao R, Chinai JM, Buhl S, Scandiuzzi L, Ray A, Jeon H, Ohaegbulam KC, Ghosh K, Zhao A, Scharff MD, Zang X (2013). HHLA2 is a member of the B7 family and inhibits human CD4 and CD8 T-cell function. Proc Natl Acad Sci USA.

[10] Arafa E, Bondzie PA, Rezazadeh K, Meyer RD, Hartsough E, Henderson JM, Schwartz JH, Chitalia V, Rahimi N (2015). TMIGD1 is a novel adhesion molecule that protects epithelial cells from oxidative cell injury. Am J Pathol.

[11] Iyer SV, Ranjan A, Elias HK, Parrales A, Sasaki H, Roy BC, Umar S, Tawfik OW, Iwakuma T (2016). Genome-wide RNAi screening identifies TMIGD3 isoform1 as a suppressor of NF-κB and osteosarcoma progression. Nat Commun.

[12] Nerlov C (2007). The C/EBP family of transcription factors: a paradigm for interaction between gene expression and proliferation control. Trends Cell Biol.

[13] Calkhoven CF, Müller C, Leutz A (2000). Translational control of C/EBPalpha and C/EBPbeta isoform expression. Genes Dev.

[14] Gomis RR, Alarcón C, Nadal C, Van Poznak C, Massagué J (2006). C/EBPbeta at the core of the TGFbeta cytostatic response and its evasion in metastatic breast cancer cells. Cancer Cell.

[15] Gao J, Aksoy BA, Dogrusoz U, Dresdner G, Gross B, Sumer SO, Sun Y, Jacobsen A, Sinha R, Larsson E, Cerami E, Sander C, Schultz N (2013). Integrative analysis of complex cancer genomics and clinical profiles using the cBioPortal. Sci Signal.

[16] Cerami E, Gao J, Dogrusoz U, Gross BE, Sumer SO, Aksoy BA, Jacobsen A, Byrne CJ, Heuer ML, Larsson E, Antipin Y, Reva B, Goldberg AP (2012). The cBio cancer genomics portal: an open platform for exploring multidimensional cancer genomics data. Cancer Discov.

[17] Nguyen-Ngoc KV, Cheung KJ, Brenot A, Shamir ER, Gray RS, Hines WC, Yaswen P, Werb Z, Ewald AJ (2012). ECM microenvironment regulates collective migration and local dissemination in normal and malignant mammary epithelium. Proc Natl Acad Sci USA.

[18] Abukhdeir AM, Park BH (2008). P21 and p27: roles in carcinogenesis and drug resistance. Expert Rev Mol Med.

[19] Han J, Sun P (2007). The pathways to tumor suppression via route p38. Trends Biochem Sci.

[20] Hui L, Bakiri L, Mairhorfer A, Schweifer N, Haslinger C, Kenner L, Komnenovic V, Scheuch H, Beug H, Wagner EF (2007). p38alpha suppresses normal and cancer cell proliferation by antagonizing the JNK-c-Jun pathway. Nat Genet.

[21] Ambrosino C, Nebreda AR (2001). Cell cycle regulation by p38 MAP kinases. Biol Cell.

[22] Todd DE, Densham RM, Molton SA, Balmanno K, Newson C, Weston CR, Garner AP, Scott L, Cook SJ (2004). ERK1/2 and p38 cooperate to induce a p21CIP1-dependent G1 cell cycle arrest. Oncogene.

[23] Alderton F, Humphrey PP, Sellers LA (2001). High-intensity p38 kinase activity is critical for p21(cip1) induction and the antiproliferative function of G(i) protein-coupled receptors. Mol Pharmacol.

[24] Saha K, Adhikary G, Kanade SR, Rorke EA, Eckert RL (2014). p38δ regulates p53 to control p21Cip1 expression in human epidermal keratinocytes. J Biol Chem.

[25] Zarubin T, Han J (2005). Activation and signaling of the p38 MAP kinase pathway. Cell Res.

[26] Bulavin DV, Saito S, Hollander MC, Sakaguchi K, Anderson CW, Appella E, Fornace AJ (1999). Phosphorylation of human p53 by p38 kinase coordinates N-terminal phosphorylation and apoptosis in response to UV radiation. EMBO J.

[27] Philipp-Staheli J, Kim KH, Liggitt D, Gurley KE, Longton G, Kemp CJ (2004). Distinct roles for p53, p27Kip1, and p21Cip1 during tumor development. Oncogene.

[28] Alao JP (2007). The regulation of cyclin D1 degradation: roles in cancer development and the potential for therapeutic invention. Mol Cancer.

[29] Kim MY, Han SI, Lim SC (2011). Roles of cyclin-dependent kinase 8 and β-catenin in the oncogenesis and progression of gastric adenocarcinoma. Int J Oncol.

[30] Delston RB, Matatall KA, Sun Y, Onken MD, Harbour JW (2011). p38 phosphorylates Rb on Ser567 by a novel, cell cycle-independent mechanism that triggers Rb-Hdm2 interaction and apoptosis. Oncogene.

[31] Gubern A, Joaquin M, Marquès M, Maseres P, Garcia-Garcia J, Amat R, González-Nuñez D, Oliva B, Real FX, de Nadal E, Posas F (2016). The N-Terminal Phosphorylation of RB by p38 Bypasses Its Inactivation by CDKs and Prevents Proliferation in Cancer Cells. Mol Cell.

[32] Lee C, Huang CH (2013). LASAGNA-Search: an integrated web tool for transcription factor binding site search and visualization. Biotechniques.

[33] Porse BT, Bryder D, Theilgaard-Mönch K, Hasemann MS, Anderson K, Damgaard I, Jacobsen SE, Nerlov C (2005). Loss of C/EBP alpha cell cycle control increases myeloid progenitor proliferation and transforms the neutrophil granulocyte lineage. J Exp Med.

[34] Ramji DP, Foka P (2002). CCAAT/enhancer-binding proteins: structure, function and regulation. Biochem J.

[35] Johnson PF (2005). Molecular stop signs: regulation of cell-cycle arrest by C/EBP transcription factors. J Cell Sci.

[36] Iakova P, Awad SS, Timchenko NA (2003). Aging reduces proliferative capacities of liver by switching pathways of C/EBPalpha growth arrest. Cell.

[37] Sebastian T, Malik R, Thomas S, Sage J, Johnson PF (2005). C/EBPbeta cooperates with RB: E2F to implement Ras(V12)-induced cellular senescence. EMBO J.

[38] Robinson GW, Johnson PF, Hennighausen L, Sterneck E (1998). The C/EBPbeta transcription factor regulates epithelial cell proliferation and differentiation in the mammary gland. Genes Dev.

[39] Timchenko NA, Wilde M, Nakanishi M, Smith JR, Darlington GJ (1996). CCAAT/enhancer-binding protein alpha (C/EBP alpha) inhibits cell proliferation through the p21 (WAF-1/CIP-1/SDI-1) protein. Genes Dev.

[40] Timchenko NA, Harris TE, Wilde M, Bilyeu TA, Burgess-Beusse BL, Finegold MJ, Darlington GJ (1997). CCAAT/enhancer binding protein alpha regulates p21 protein and hepatocyte proliferation in newborn mice. Mol Cell Biol.

[41] Buck M, Turler H, Chojkier M (1994). LAP (NF-IL-6), a tissue-specific transcriptional activator, is an inhibitor of hepatoma cell proliferation. EMBO J.

[42] Zhu S, Oh HS, Shim M, Sterneck E, Johnson PF, Smart RC (1999). C/EBPbeta modulates the early events of keratinocyte differentiation involving growth arrest and keratin 1 and keratin 10 expression. Mol Cell Biol.

[43] Cattaneo E, Laczko E, Buffoli F, Zorzi F, Bianco MA, Menigatti M, Bartosova Z, Haider R, Helmchen B, Sabates-Bellver J, Tiwari A, Jiricny J, Marra G (2011). Preinvasive colorectal lesion transcriptomes correlate with endoscopic morphology (polypoid vs. nonpolypoid). EMBO Mol Med.

[44] Sherr CJ, Roberts JM (1999). CDK inhibitors: positive and negative regulators of G1-phase progression. Genes Dev.

[45] Chu IM, Hengst L, Slingerland JM (2008). The Cdk inhibitor p27 in human cancer: prognostic potential and relevance to anticancer therapy. Nat Rev Cancer.

[46] James MK, Ray A, Leznova D, Blain SW (2008). Differential modification of p27Kip1 controls its cyclin D-cdk4 inhibitory activity. Mol Cell Biol.

[47] Noseda M, Chang L, McLean G, Grim JE, Clurman BE, Smith LL, Karsan A (2004). Notch activation induces endothelial cell cycle arrest and participates in contact inhibition: role of p21Cip1 repression. Mol Cell Biol.

[48] Rahimi N, Dayanir V, Lashkari K (2000). Receptor chimeras indicate that the vascular endothelial growth factor receptor-1 (VEGFR-1) modulates mitogenic activity of VEGFR-2 in endothelial cells. J Biol Chem.

[49] Wang YK, Wang YH, Wang CZ, Sung JM, Chiu WT, Lin SH, Chang YH, Tang MJ (2003). Rigidity of collagen fibrils controls collagen gel-induced down-regulation of focal adhesion complex proteins mediated by alpha2beta1 integrin. J Biol Chem.

