# Supplementary material for: TMIGD1 acts as a tumor suppressor through regulation of p21Cip1/p27Kip1 in renal cancer
Source: Oncotarget. 2017 Dec 26;9(11):9672–84. doi: 10.18632/oncotarget.23822 (PMC5839393; doi:10.18632/oncotarget.23822)
Supplement: Supplementary file 1 [file oncotarget-09-9672-s001.pdf]

TMIGD1 acts as a tumor suppressor through regulation of p21Cip1/p27Kip1 in renal Cancer

SUPPLEMENTARY MATERIALS

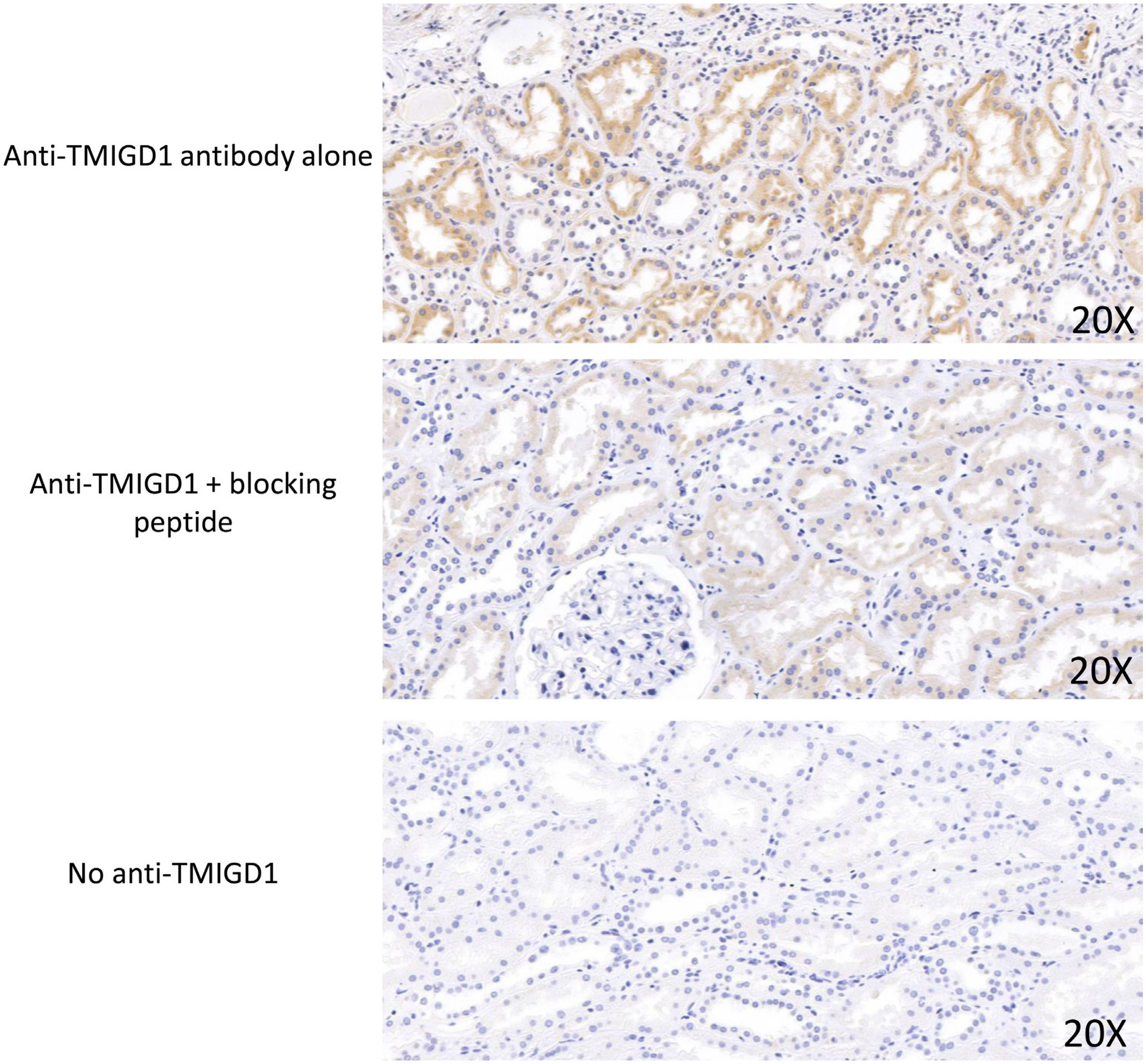

Supplementary Figure 1: Validation of TMIGD1 antibody. Anti-TMIGD1 antibody (1:750) was used to stain human kidney tissue or it pre-incubated with the blocking TMIGD1 peptide (10X) for 1 hour before use for immunohistochemistry. Also, shown is the human kidney tissue subjected to IHC staining without anti-TMIGD1 antibody.

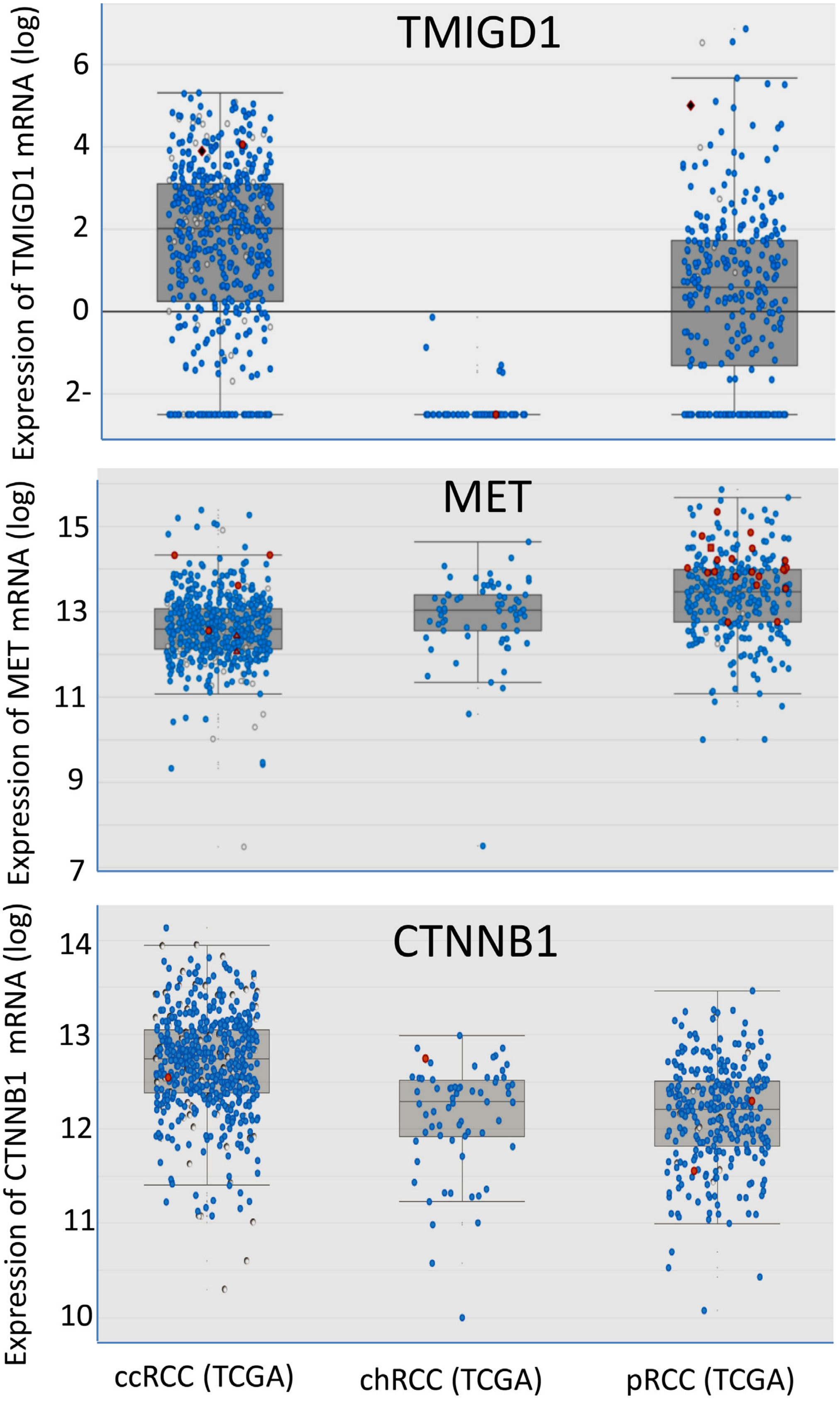

Supplementary Figure 2: Analysis of TMIGD1, MET and ß-catenin expression in renal cancer based on the TCGA data using cBioPortal for Cancer Genomics (http://cbioportal.org). The data shows TMIGD1 expression is downregulated in the three major RCC cancer types. Chromophobe RCC (chRCC) Papillary RCC (pRCC) and clear cell RCC (ccRCC). Expression of both Met and ß-catenin (CTNNB1) were upregulated.

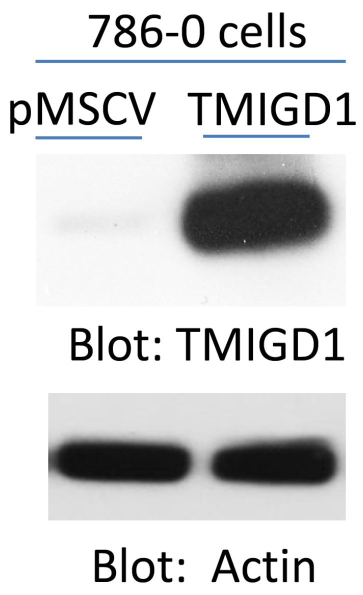

Supplementary Figure 3: Ectopic expression of TMIGD1 in 786-0 cells. 786-0 cells expressing an empty retroviral vector (EV) or TMIGD1 were lysed and whole cell lysates was blotted for TMIGD1 or for a loading control protein, actin.

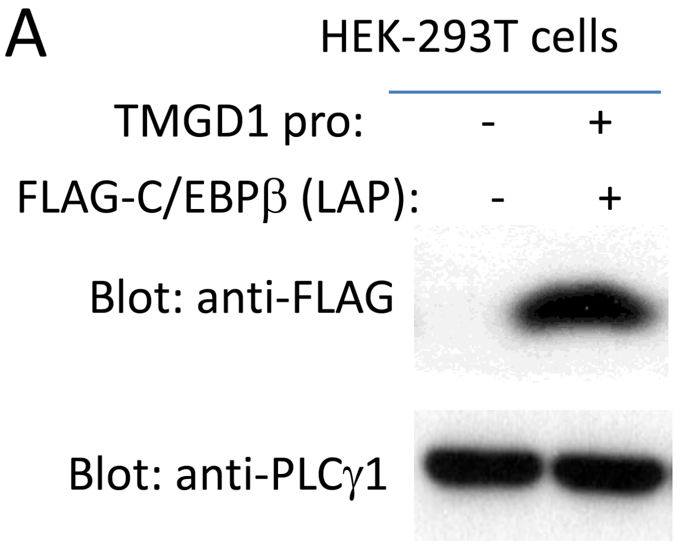

Supplementary Figure 4: Expression of C/EBPß (LAP) in HEK-293 cells. HEK-293 cells were co-transfected with the full-length TMIGD1 promotor construct alone or with C/EBPß (LAP) as indicated. Cells were lysed after 48hrs and whole cell lysates were blotted for LAP using anti-FLAG antibody as indicated.

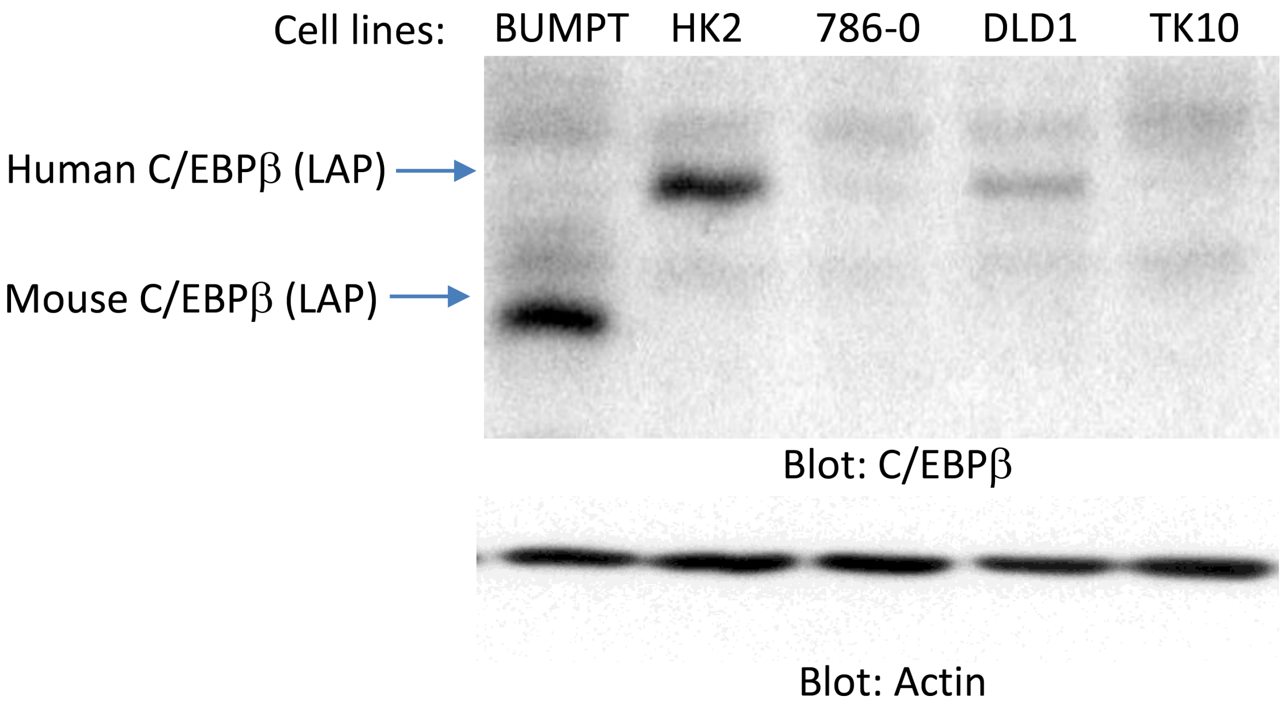

Supplementary Figure 5: Expression of C/EBPß in normal and cancer renal cell lines. (A) Whole cell lysates derived from human (HK2) and mouse (BUMPT) normal renal epithelial cells and renal tumor cell lines subjected to western blot analysis using anti-C/EBPß antibody (1:1000) or for a loading control, actin.

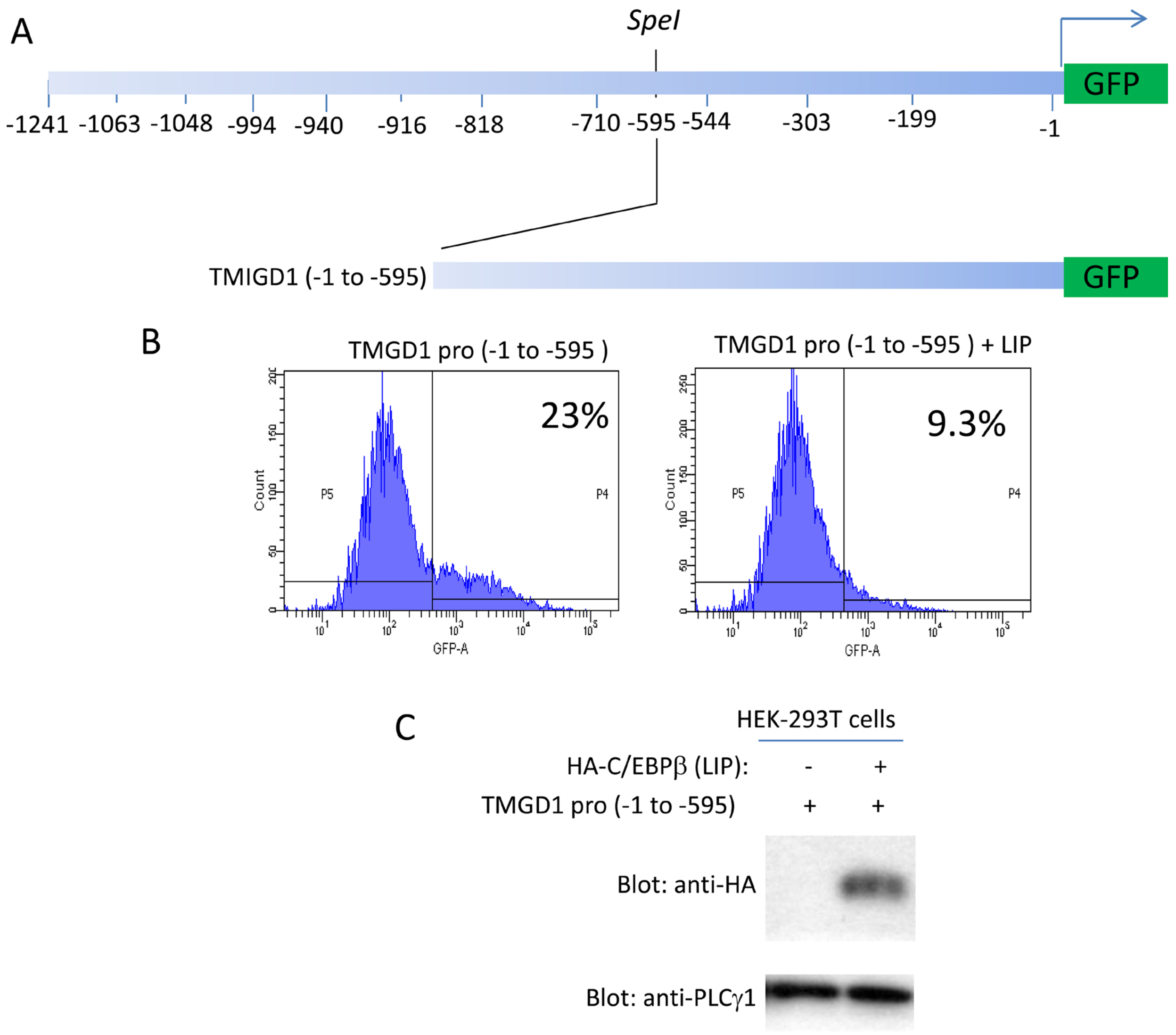

Supplementary Figure 6: Transcriptionally inactive C/EBPß (LIP) inhibits the promoter activity of TMIGD1. (**A**) Schematic of full-length and 5'-truncated TMIGD1 (-1 to -595) promoter is shown. (**B**) HEK-293T cells transfected with TMIGD1 (-1 to -595) promoter alone or with C/EBPß (LIP). After 48 hours cells prepared and GFP expression was analyzed by FACS analysis. The same cells were lysed and blotted with expression of C/EBPß (LIP), using HA antibody.

MATERIALS AND METHODS

Antibodies, plasmids and primers

The following antibodies were purchased from Cell Signaling. C/EBPß antibody (#3082), anti-p21 antibody (DCS60), anti-p27 antibody (D37H1), anti-phospho-p38 antibody (Thr180/Tyr182) Antibody #9211), phospho-Rb antibody (D20B12), phospho-CylinD1 antibody (D29B3), Phospho-Cd2 antibody (10A11). TMIGD1 was cloned into pMSCV. puro vector (Clontech) as previously described (8). TMIGD1 promoter (1.24 kb) was PCR amplified from the mRNA of human renal epithelial (HK2) cells using primers (sense primer 5′GTCTAACTAGTGTGCGGCAGATTCACATGCT3′ and anti-sense primer 5′ CAATGCGGCCGCCTTTCCCTC CACTCCACCGAG3′) and subsequently cloned into pHAGE-GFP (Zs Green) reporter vector (kindly provided by Dr. George Murphy, Boston University). The 1.24kb TMIGD1 promoter was used as a template to create additional truncated forms of TMIGD1 promoter. All the constructs were sequenced to confirm their sequence identities. pcDNA3 Flag C/EBPß/LAP (Plasmid #66979) (21) and pCMV-HA C/EBPß/LIP (Plasmid #15739) (19) were purchased from Addgene.

Electrophoretic mobility shift assay (EMSA) assay

Cell lysate from HEK-293T cells expressing C/EBPβ (LAP)-FLAG was used as a source of C/EBPβ. Biotin-labeled TMIGD1 promotor probe (-595 to -1) was generated by PCR using 5' biotinylated primer was incubated with or without 1µl of lysate under the presence of Poly (dI•dC) for 20 min in room temperature. 5µl of unlabeled probe was added into the negative control group. 4.5% of polyacrylamide gel in 0.5X TBE was prepared and pre-ran for 30 min. After electrophoresis, the gel was transferred to a PVDF membrane. The membrane was fixed by UV and probed with FLAG or biotin antibody.

Branching-morphogenesis assay

786-0 cells expressing either empty vector or TMIGD1 were subjected to branching-morphogenesis as described with some modifications (15). Briefly, collagen (9 volumes of collagen type I, Corning, Bedford, MA) was mixed with 5.7X RPMI (1volume), 2.5% NaHCO3 (0.5 volume), 0.1M HEPES (1 volume), 0.17 M CaCl2 (0.1 volume), 1 N NaOH (0.1 volume), and 4.3 volumes of cells in culture medium (RPMI plus 10% FBS) on ice. The mixture (1ml) containing cells (1×10^5) and collagen mix was plated in 35-mm plates and kept in an incubator (5% CO2 in air, 37°C) to allow for the gelation. After 2 hours gelation, plates were overlaid with 1.5 ml of culture medium (RPMI plus 10% FBS) which was then replaced every other day.
